# VEGF-A in Cardiomyocytes and Heart Diseases

**DOI:** 10.3390/ijms21155294

**Published:** 2020-07-26

**Authors:** Mariantonia Braile, Simone Marcella, Leonardo Cristinziano, Maria Rosaria Galdiero, Luca Modestino, Anne Lise Ferrara, Gilda Varricchi, Giancarlo Marone, Stefania Loffredo

**Affiliations:** 1 Department of Translational Medical Sciences and Center for Basic and Clinical Immunology Research (CISI), University of Naples Federico II, 80131 Naples, Italy; brailemariantonia@gmail.com (M.B.); s.marcella92@gmail.com (S.M.); l.cristinziano@gmail.com (L.C.); mrgaldiero@libero.it (M.R.G.); modestino.luca@gmail.com (L.M.); anneliseferrara@gmail.com (A.L.F.); gildanet@gmail.com (G.V.); 2 WAO Center of Excellence, 80131 Naples, Italy; 3 CNR Institute of Experimental Endocrinology and Oncology “G. Salvatore”, 80131 Naples, Italy; 4 Department of Public Health, University of Naples Federico II, 80131 Naples, Italy; gcmarone@hotmail.it; 5 Azienda Ospedaliera Ospedali dei Colli – Monaldi Hospital Pharmacy, 80131 Naples, Italy

**Keywords:** angiogenesis, atherosclerosis, cardiovascular disease, inflammation, ischemic heart disease, myocardial infarction

## Abstract

The vascular endothelial growth factor (VEGF), a homodimeric vasoactive glycoprotein, is the key mediator of angiogenesis. Angiogenesis, the formation of new blood vessels, is responsible for a wide variety of physio/pathological processes, including cardiovascular diseases (CVD). Cardiomyocytes (CM), the main cell type present in the heart, are the source and target of VEGF-A and express its receptors, VEGFR1 and VEGFR2, on their cell surface. The relationship between VEGF-A and the heart is double-sided. On the one hand, VEGF-A activates CM, inducing morphogenesis, contractility and wound healing. On the other hand, VEGF-A is produced by CM during inflammation, mechanical stress and cytokine stimulation. Moreover, high concentrations of VEGF-A have been found in patients affected by different CVD, and are often correlated with an unfavorable prognosis and disease severity. In this review, we summarized the current knowledge about the expression and effects of VEGF-A on CM and the role of VEGF-A in CVD, which are the most important cause of disability and premature death worldwide. Based on clinical studies on angiogenesis therapy conducted to date, it is possible to think that the control of angiogenesis and VEGF-A can lead to better quality and span of life of patients with heart disease.

## 1. Vascular Endothelial Growth Factor

Vascular endothelial growth factor (VEGF) has been discovered to be a permeability-enhancing agent leading to the disruption of intercellular contacts and the increase of permeability [1]. VEGF family, in humans, consists of five separate gene products: VEGF-A, VEGF-B and Placental growth factor, which are key regulators of blood vessel growth; and VEGF-C and VEGF-D, which modulate lymphangiogenesis [2]. VEGF-A, a homodimeric glycoprotein of approximately 45 kDa [3], is the key to vasculogenesis (the *de novo* formation of vessels) and angiogenesis (the formation of new vessels from preformed vasculature) [4,5]. VEGF-A induces cellular chemotaxis [6] and the expression of plasminogen activators [7] and collagenases [8] in endothelial cells (EC). VEGF-A promotes blood vessel growth and remodeling processes, and it also provides survival and mitogenic stimuli for EC [9,10,11]. During inflammation and tumorigenesis, sequestered VEGF can be released by proteases including matrix metalloproteinases [12,13], plasmin, urokinase-type plasminogen activator, elastase and tissue kallikrein. These proteases, affecting VEGF-A activity through VEGF-A cleavage, activation and degradation, can both promote angiogenesis, for example as a key step in carcinogenesis, and suppress VEGF’s angiogenic effects [14].

In humans, VEGF-A gene locus is on chromosome 6p21.1 and contains eight exons and seven introns. Through alternative splicing, human VEGF-A is represented by multiple isoforms [15,16]. The four major expressed variants are 121, 165, 189 and 206 amino acids long, with the VEGF-A_165_ representing the predominant species of VEGF-A [2,17,18,19,20]. The larger isoforms, VEGF-A_165_, VEGF-A_189_ and VEGF-A_206_, are basic and bind to isolated heparin and heparin proteoglycans distributed on cellular surfaces and extracellular matrices [21,22]. The VEGF short form, VEGF-A_121_, is acidic and is more freely diffusible [20,21]. VEGF gene expression is upregulated by a variety of factors, such as growth factors (i.e., fibroblast growth factors, epidermal growth factor, tumor necrosis factor (TNF) [23,24,25,26,27,28].

VEGF isoforms, with their differences in biotransport, sequestration and receptor binding, induce a spectrum of vascular phenotypes [20] from the malformed, edematous, hypovascular networks of VEGF-A_120_ (VEGF-A_121_ in human) due to dilated and poorly branched vessel generation [29] to the stable, thin and branching vessels of VEGF-A_188_ (VEGF-A_189_ in human) [30]. These vascular phenotypes are related to the ability of VEGF-A isoforms to induce the sprouting, migratory phenotype. VEGF-A_165_ (VEGF-A_164_ in mouse) is the first isoform characterized and as a potent stimulator of angiogenesis [20]. Its expression is adequate neonatal growth and has been well documented in tissues during physiological and/or pathological conditions [2,17].

The physiological effects of VEGF-A are driven by the binding to two homologous VEGF-A receptors. These receptors are known as VEGFR1 (Flt-1 in mice) and VEGFR2 (Flk-1; KDR) [31,32]. They areencoded by separate genes and are members of the class IV receptor tyrosine kinase family [33]. The expression of VEGFR has been demonstrated on EC, macrophages, mast cells and smooth muscle cells [34,35,36,37]. Although VEGFR1 binds VEGF-A with high affinity, it is believed to act primarily by modulating the availability of VEGF-A for binding to VEGFR2 [38,39,40,41]. Receptors for the VEGF-A, apart from those bound to cellular membranes, also occur in soluble form [42]. The soluble VEGFR1 (sVEGFR1) and sVEGFR2 receptors, binding VEGF-A before it reaches membrane-bound receptors on surface cells, are regarded as physiological inhibitors of the processes of angiogenesis and neoangiogenesis [43].

## 2. Cardiomyocytes

Cardiomyocytes (CM), also known as myocardiocytes or cardiac myocytes, are the muscle cells that belong to the heart muscle [44]. CM are contractile cells that, through their autorhythmicity and coordinated action with other CM, enable the heart to function as a pump [45]. This mechanism ensures that sufficient oxygenated blood and metabolites reach the tissues to meet the body’s needs, whether at rest or during exercise [46]. To fulfill these functions, the CMare equipped with highly specialized subcellular machinery [44,47]. The first one of this system is the basement membrane, whose functions are to separate the intracellular structure from the extracellular environment, promote the exchange of macromolecules and catch ions, such as calcium [48,49,50].

A specialized structure of the CM is the sarcolemma. The sarcolemma controls the type of molecules that enters the cell [51,52] and guarantees the contraction and relaxation of the cells [53,54]. The CM cytoskeleton forms an important structural link between the extracellular environment and the contractile apparatus, and it can influence CM geometry and function through the phosphorylation of some of its proteins [55]. Myofilaments, contractile proteins formed from myosin and actin, are additional important subcellular structures. The interaction of these motor proteins guarantees the contraction of the muscle that allows blood to be pumped throughout the body [56,57,58]. Finally, CM contain a large number of mitochondria, which maintain high levels of ATP required by the cells to promote the contraction and relaxation of the heart muscle [59,60].

CM produce and secrete several mediators such as adiponectin [61], regenerating islet-derived protein 3-β [62], TNF-α, interleukin-1β (IL-1β), interferon γ [63], transforming growth factor β (TGF-β) [64], IL-6 and IL-11 [65,66]. It has been shown that CM are also a source of angiogenic factors, such as angiopoietin 1 (ANGPT1), ANGPT2 [67,68] and mainly VEGF-A [69].

## 3. Cardiomyocytes as Producers of VEGF-A

CM are a source of VEGF-A, which performs several functions in the heart. VEGF-A is the main regulator of vascular permeability and of angiogenesis [70]. In the mouse model, CM-specific deletion of VEGF-A alters negatively vasculogenesis/angiogenesis and causes a thinner ventricular wall [71], confirming mutual signaling from the CM to the EC during heart development. Interestingly, in mice lacking the VEGF-A the myocardial microvasculature is underdeveloped, but the coronary artery structure is preserved, involving a different signaling pathway for vasculogenesis/angiogenesis in the myocardium and epicardial coronary arteries [72]. Mice with CM-specific deletion of VEGF-A_164_ and VEGF-A_188_ isoforms show impaired myocardial angiogenesis with subsequent ischemic cardiomyopathy and heart failure [73]. These mice also exhibit heart capillaries that are more irregular, tortuous and dilated, indicating an incomplete vessel remodeling. Therefore, VEGF-A induces myocardiac angiogenesis and enhances vascular permeability and EC proliferation [73].

In mouse model, the production of VEGF-A from CM also blocks the transformation of cardiac endocardial into mesenchymal [74]. This mechanism plays an important role in cardiac cushion formation and requires gentle control of VEGF-A concentration [74,75,76]. In the same animal model, low levels of VEGF-A induce the transformation of cardiac endocardial into mesenchymal, whereas high levels of VEGF-A block this transformation [77]. Interestingly, this signal induced by the production of VEGF-A from CM for endocardial–mesenchymal transformation may be regulated by an endothelial-derived feedback process through the calcineurin/NFAT pathway [78], demonstrating the importance of EC–CM interactions for cardiac morphogenesis [67].

In rat CM, mechanical stress regulates VEGF-A expression, while stretch improves the secretion [79]. Factors involved in the upregulation of VEGF-A expression in mice hypertrophied CM include hypoxia-inducible factor 1-α [80,81,82], but also NFκB [83], TGF-β [84] and endothelin-1 [85]. Mice with CM lacking GATA4, a transcription factor that directly binds the VEGF-A promoter, show a poor capillary density in their hearts, while overexpression of GATA4 markedly improves cardiac vascularization and function following myocardial infarction by promoting angiogenesis (via VEGF-A production), hypertrophy, and inhibiting apoptosis [86,87]. In addition to GATA4, GATA6 and GATA2 also control angiogenesis [88,89,90]. In CM, the activation p38MAPK induces VEGF-A production, whereas its secretion occurs in an Sp1-dependent manner [91,92]. In this regard, the heart of mice with p38-inactivated CM exhibits compromised compensatory angiogenesis after pressure overload, and it is prone to early onset of heart failure. In summary, p38αMAPK plays a critical role in the cross dialogue between CM and vascularization by regulating stress-induced VEGF-A expression and secretion in murine CM [93].

To date, there are no data on the expression and release of VEGF in human CM. Further studies are required to define the mechanistic role of VEGF-A in humans and whether this knowledge may be beneficial in specific patient populations.

**Figure 1:**
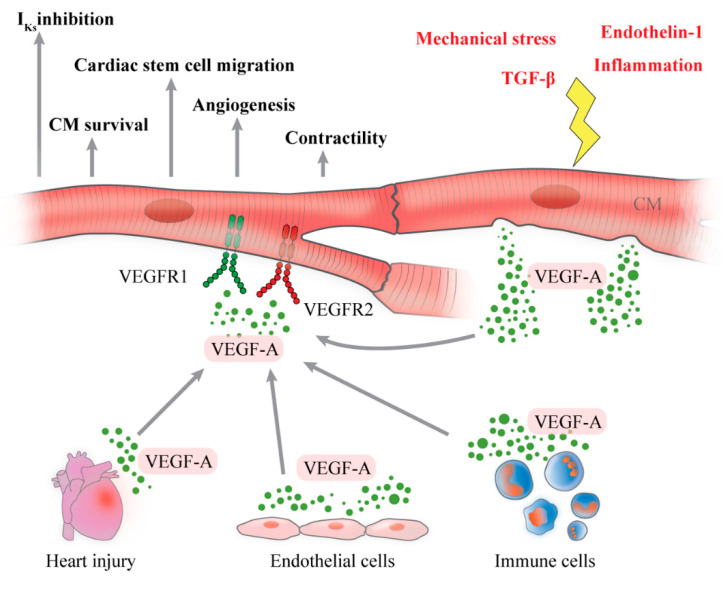
Schematic representation of cardiomyocytes (CM) as source and target of vascular endothelial growth factor-A (VEGF-A). A plethora of stimuli including inflammation, mechanical stress, endothelin-1 and transforming growth factor-β (TGF-β) induce CM to produce and release VEGF-A, whose function is to promote angiogenesis in myocardial tissue. CM are also a target of VEGF-A, produced by several cells and during heart injury, through binding with VEGFR1 and VEGFR2, expressed on their surface. CM activation, induced by VEGF-A, enhances CM survival, contractility, cardiac stem cell recruitment, cardiac angiogenesis and reduction of potassium current (I_Ks_).

The release of VEGF-A by CM is schematized in Figure 1.

## 4. Cardiomyocytes as Target of VEGF-A

CM are both producers and targets of VEGF-A [94,95,96]. This latter exhibits a plethora of actions in reparative wound healing within the myocardium, including vasculogenesis [97], recruitment and homing of stem cells [98], decreased apoptosis [99,100] and increased vasodilatation [101] and modulation of the autonomic response [102]. As previously described, VEGF-A exerts its biological effects by interacting with two main tyrosine kinase receptors, VEGFR1 and VEGFR2, with an affinity for VEGFR1 higher than VEGFR2 [97,103]. VEGFR1 and VEGFR2 are both expressed on CM surface, but VEGFR1 is upregulated following hypoxia and oxidative stress [104]. VEGFR1 is also important for cardiac contractility; in fact, the heart uses VEGF-A–phospholipase Cγ1 signaling to control the strength of the heartbeat [105,106]. VEGFR2 is largely expressed on the surviving mouse CM after acute myocardial infarction, and CM viability is significantly improved with VEGF-A_164_ [107].

It has been demonstrated in the rat model that VEGF-A inhibits CM apoptosis and activates the expression of genes involved in myocardial contractility and metabolism [104,108]. VEGF-A involvement in heart repair also encourages cardiac stem cell migration via the PI3K/Akt pathway [98]. The same research group demonstrates that the pathway VEGF-A together with myocardial stromal cell-derived factor-1 (SDF-1) encourages cardiac stem cell mobilization and myocardial repair within infarcted heart [109,110].

VEGF-A plays a pivotal role in triggering the cardiac angiogenic response following acute infarcted myocardium [111]. VEGF-A and VEGFR expression is increased at the border zone only in the first day post rat myocardial infarction, but not in the later stages [112], and then it is suppressed during the first week when angiogenesis is more active [113]. Therefore, the withdrawal of VEGF-A may be also related to the later vascular stabilization in the infarcted myocardium [114]. Moreover, VEGF-A reduces potassium current (I_Ks_) through a phosphatidylinositol 3-kinase–mediated molecular pathway, increasing the duration of cardiac action potential in guinea pig ventricular CM. In conclusion, we speculate that VEGF-A, after being released by CM, may have autocrine/paracrine effects activating CM by binding to VEGFRs.

The effects of VEGF-A on CM are shown in Figure 1.

## 5. VEGF-A and Angiogenesis in Cardiovascular Diseases

As previously stated, angiogenesis is the process of formation of new blood vessels from pre-existing vessels, involving cell proliferation, migration, differentiation, tube formation and regulation of angiogenic factors [115]. It is responsible for a great variety of physiological and pathological processes, including cardiovascular diseases (CVD) [116].

CVD are pathological processes representing the number one cause of mortality in the world [117]. Among them, the most recognized CVD are ischemic disease and atherosclerosis [118,119], which are the most important cause of disability and premature death worldwide [117]. Therefore, CVD seriously affects the quality of life, increasing the psychological and economic burden [117,120].

### 5.1. Ischemic Heart Disease

Ischemic heart disease (IHD) remains the leading cause of death worldwide [121]. World Health Organization reports that 740 million people die of IHD annually all around the world, accounting for the death of 13.2% of the total population [Organization WH. World Health Organization report. May 2014, http://www.who.int/mediacentre/factsheets/fs310/zh/]. Myocardial infarction (MI) is one of the main manifestations of IHD, which induces myocardial necrosis or apoptosis in a short time [122], leading to heart failure with a poor prognosis [123]. It has been classified as the main cause of death in IHD [124]. To date, there are several types of treatments for MI, such as reducing incidence of coronary atherosclerosis [125], antithrombotic therapy including vitamin K antagonists [126], antiplatelet therapy with low-dose aspirin [127] and clopidogrel [128]. In the last years, the therapeutic angiogenesis has been proposed as a new strategy for the treatment of MI. Angiogenesis appears in all vascularized organs [129]. Although ischemia leads to endogenous myocardial angiogenesis, it cannot reach the effect to maintain normal capillary density [130]. Experiments conducted on rat models reveal that serum VEGF-A levels are positively associated with increased microvessel density in the infarcted area, suggesting VEGF-A role in myocardial remodeling and angiogenesis [131,132,133,134,135]. There is compelling evidence that patients with MI have high serum levels of VEGF-A [136]. Moreover, altered levels of VEGF-A are detected also in plasma after MI and are correlated with high inflammation cytokine concentrations [137] suggesting that increased levels of VEGF-A are a part of ongoing inflammatory activity. Since high concentrations of VEGF-A in these patients lead to neovascularization of inflamed plaques and their destabilization, VEGF-A levels are a negative prognostic value [137]. Therefore, therapeutic stimulation of angiogenesis has been regarded as an effective treatment for IHD [138]. Stroke is another main manifestation of IHD. In stroke, the breakdown of the blood–brain barrier leads to the release of reactive oxygen species capable of transforming astrocytes into reactive astrocytes. These reactive astrocytes modify the extracellular matrix (ECM) [139] with a consequent restructuring of the ECM with the formation of ECM traits [140]. EC use these new ECM traits to establish new capillary buds [141]. This mechanism is strictly regulated by a balance between proangiogenic and angiostatic factors [142,143,144,145]. At the onset of ischemia, the combined presence of nitric oxide (NO) and VEGF-A leads to vasodilation and an increase in vascular permeability. This condition generates the extravasation of plasma proteins that promote temporary communication for the migration of EC to ensure vascular germination [146], with subsequent dissociation of smooth muscle cells and the loosening of the ECM. ANGPT2, an inhibitor of Tie2 signaling, and matrix metallopeptidases regulate these mechanisms [117]. After the germination path has been established, the EC proliferate and migrate under VEGF-A signaling, and new blood vessels are maintained by ANGPT1 by activating the Tie2 receptor [147]. To date, understanding the dynamic changes of these angiogenic factors after stroke could be useful for developing effective therapeutic strategies. In this regard, high levels of VEGF-A within hours of a stroke are correlated with angiogenesis in the injured area of the brain. Administration of VEGF-A, after a few minutes of reoxygenation following hypoxic ischemia, exhibits a reduction in brain injury in rats [117].

### 5.2. Atherosclerosis

Atherosclerosis is an alteration characterized by the accumulation of cholesterol and non-resolving inflammation in the vascular wall of the medium and large arteries [148]. Neovascularization in atherosclerotic damage is essential for plaque growth and instability [149]. In atherosclerosis, VEGF-A performs a dual function [150]. On the one hand, it induces beneficial effects, protecting the EC by increasing the expression levels of anti-apoptotic proteins and NO synthesis [2]. On the other hand, VEGF-A induces harmful effects, acting as mitogen by re-endothelialization [151] and prevention or repair of the endothelial lesion that can induce atherogenesis [152]. In addition, VEGF-A promotes monocyte adhesion, transendothelial migration and activation [153,154], improving also endothelium permeability [1], adhesion protein expression [155] and monocyte chemoattractant protein-1 [156]. In human coronaries, VEGF-A and its receptors are not found in normal coronary segments, but their expression increases in EC of microcapillaries, in macrophages and in partially differentiated smooth muscle cells of atherosclerotic lesions [157]. VEGF-A is identified as a marker of atherosclerosis, performing experiments on rabbits [158,159]. Chronic stress reduces atherosclerosis tunica media and induces plaque instability, promoting angiogenesis by release of VEGF-A identified in a great amount in serum [158]. Administration of Resveratrol decreases VEGF-A serum concentration, reducing formation and evolution of atherosclerotic lesions in rabbit model [159]. It has been demonstrated that anti-angiogenic factors reduce atherosclerosis development in various animal models [160]. Therefore, clinical trials with anti-angiogenic drugs such as anti-VEGF/VEGFR, used in anti-cancer therapy, show cardiovascular adverse effects and require additional investigations [161]. Conversely, plasma VEGF-A is weakly associated with cardiovascular risk factors, suggesting circulating VEGF-A has only a little influence on the development of atherosclerosis [162].

As a result of atherosclerosis, there are two other important pathological processes: Atherothrombosis and coronary artery disease.

Atherothrombosis is a complex inflammatory pathological process that involves lipid deposition in the arterial wall with recruitment of circulating leukocytes [163]. This continuous accumulation leads to the growth of a plaque that could become unstable and break, triggering the formation of a thrombus [164]. An occlusive thrombus may possibly be responsible for an ischemic event [165]. In this process, inflammation plays an important role in all phases, especially the reactive protein C, which is one of the main ones responsible for the cascade of events that induces thrombosis [166].

The VEGF-A signaling pathway (VSP) plays an important role in EC, and its inhibition has huge effects on thrombosis [114,167]. Generalized endothelial dysfunction predisposes to both arterial and venous thrombosis [168]. VSP blockade induces vascular toxicity, including arterial thromboembolic events (ATE) [169,170,171]. VSP inhibitors are antibodies, acting directly on VEGF-A, such as bevacizumab [172] and tyrosine kinases inhibitors [173], binding to the kinase domain of VEGF-A receptors, such as sunintib [174] and sorafenib [161]. Age over 65 years, previous thromboembolic events, history of atherosclerotic disease and duration of VSP inhibitor therapy are possible risk factors for ATE during VSP inhibitor therapy [175].

Coronary artery disease (CAD) is a pathological process, generally caused by atherosclerosis, in which the coronary arteries are constricted or blocked. The relationship between angiogenesis and CAD is complex. Angiogenesis promotes growth [176] and vulnerability of plaques [177,178], causing intraplaque hemorrhage [179] and the influx of inflammatory cells and erythrocytes [180]. The growth of this accumulation could break the plaque, worsening the pathological condition. In this regard, the blockade of intraplaque angiogenesis is considered as a potential therapeutic target for CAD [160,181,182].

Moreover, patients with CAD have increased serum and plasma levels of VEGF-A [183,184] that correlate with IL-18 concentrations [185], a cytokine that induces VEGF-A expression [183]. This increase may indicate that VEGF-A can be considered as a marker for revascularization when coronary artery injury is critical [186]. Therefore, therapeutic angiogenesis is used to improve the ischemic myocardial reperfusion in patients with CAD and to expand the myocardial microvascular network. To date, there are several therapies. The first one is the administration of angiogenic growth factors directly (protein therapy) [187,188,189]; the second strategy is promoting angiogenic genes expression in vivo (gene therapy) [190,191,192]. Finally, there are other strategies, such as delivering stem cells (cell therapy) [193,194,195,196] or exosomes (cell-free therapy) [143,197,198,199,200].

The effects of VEGF-A in CVD are summarized in Table 1.

## 6. Conclusions

Angiogenesis is a process responsible for a great variety of physiological and pathological mechanisms, including heart diseases. VEGF-A is the key regulator of angiogenesis. In this review, we discussed present knowledge on the expression and effects on VEGF-A on cardiomyocytes and on the role of VEGF-A in CVD that are the most important cause of disability and premature death worldwide.

CM, the main cell type found in the heart, produce and release VEGF-A and express its receptors, VEGFR1 and VEGFR2, on their cell surface. The lack of VEGF-A expression in CM affects myocardial angiogenesis, resulting in conditions that impair heart functions. Moreover, CM is also a target of VEGF-A because it influences CM biology such as mobilization.

VEGF-A plays an important role in cardiac morphogenesis, cardiac contractility and wound healing within the myocardium. At the same time, high concentrations of VEGF-A are detected in several CVD and are often associated with poor prognosis and disease severity. Further scientific advances have led to the discovery of a broad range of therapeutical targets and novel biomarkers associated with cardiovascular risks. In addition to protein therapy, gene therapy, cell therapy and cell-less therapy, other concepts have been applied to this field with a large amount of energy. As our understanding of pathophisiological mechanisms of CVD becomes more refined, therapeutic angiogenesis is increasingly interesting in the resolution of these pathologies. Based on clinical trials of angiogenesis therapy conducted to date, some of the approaches appear to have modest effects and continue to be investigated, while some of them have affirmed strong evidence of its success.

In this review, we summarized interesting and thought-provoking studies implicating the role of VEGF-A in various cardiovascular diseases. We suggest that with properly designed and conducted clinical trials, it is possible to think that therapeutic angiogenesis could lead to better quality and life span of patients affected by heart diseases.

## Figures and Tables

**Table 1:** Schematic representation of VEGF-A expression in cardiovascular diseases.

Cardiovascular Diseases	VEGF-A	Effects	References
Ischemic heart disease: →Myocardial infarction	↑	microvessel density in the infarcted area, myocardial remodeling angiogenesis, neovascularization, destabilization of inflamed plaques.	[131,132,133,134,135,136,137]
→Stroke	↑	vasodilation, vascular permeability, endothelial cells migration, dissociation of smooth muscle cells, loosening of the extracellular matrix.	[146]
Atherosclerosis: →Atherothrombosis	↓	vascular toxicity, arterial thrombosis.	[169,170,171]
→Coronary artery disease	↑	angiogenesis, growth and vulnerability of plaques, intraplaque hemorrhage, inflammatory cell and erythrocyte recruitment, coronary artery revascularization.	[176,177,178,179,180]

## Abbreviations

ANGPT	angiopoietin
ATE	arterial thromboembolic events
CM	cardiomyocytes
CVD	cardiovascular disease
CAD	coronary artery disease
EC	endothelial cells
ECM	extracellular matrix
IL-	interleukin
IHD	ischemic heart disease
MI	myocardial infarction
NO	nitric oxide
sVEGF-A	soluble vascular endothelial growth factor receptor
TGF-β	transforming growth factor β
TNF	tumor necrosis factor
VEGF	vascular endothelial growth
VSP	VEGF-A signaling pathway
VEGFR	vascular endothelial growth factor receptor
